# Impact of exercise-induced alterations on gut microbiota diversity and composition: comparing effects of different training modalities

**DOI:** 10.1186/s13619-025-00244-y

**Published:** 2025-07-02

**Authors:** Yihan Wang, Shuang Bai, Tiance Yang, Jianjun Guo, Xiaoming Zhu, Ying Dong

**Affiliations:** 1 Class of Clinical Medicine, School of Basic Medical Sciences, Capital Medical University, Beijing, 100069 China; 2 School of The Third Clinical Medical College, Capital Medical University, Beijing, 100020 China; 3 Heart Center & Beijing Key Laboratory of Hypertension, Beijing Chaoyang Hospital, Capital Medical University, Beijing, 100020 China; 4 School of Kinesiology and Health, Capital University of Physical Education and Sports, Beijing, 100191 China; 5 Medical Research Center, Beijing Chaoyang Hospital and Beijing Institute of Respiratory Medicine, Capital Medical University, Beijing, 100020 China

**Keywords:** Gut Microbiota, HIFT, HIIT, MICT, 16S rRNA

## Abstract

Exercise has been shown to influence gut microbiota composition, but the specific effects of different exercise modalities on microbial diversity remain unclear. Understanding these differences is essential for optimizing exercise programs to enhance both physical fitness and gut health. This study compared the gut microbiota profiles of participants undergoing moderate-intensity continuous training (MICT), high-intensity interval training (HIIT), and high-intensity functional training (HIFT) using 16S rRNA gene sequencing. Thirty-one previously untrained healthy university students were randomly assigned into MICT (*n* = 7), HIIT (*n* = 12) and HIFT (*n* = 12). The results revealed that distinct gut microbiome profiles in participants under different exercise modes. Notably, the alpha-diversity gradually increased from the MICT group to the HIFT group. In addition, there was a progressive shift towards a Faecalibacterium-dominated microbial type from HIIT to HIFT group compared to MICT group. Individuals in the HIFT group were identified to be enriched with *Lactobacillus* and *Limosilactobacillus*, along with reduced *Actinomyces* and *Anaeromassilibacillus*. Functionally, the KEGG pathway and enzyme analysis using PICRUST2 revealed that the HIFT group exhibited prominence in muscle function-related pathways and enzymes, specifically ko00280 (valine, leucine, and isoleucine degradation), as well as the enzyme EC: 3.4.11.14 (alanine aminopeptidase). In conclusion, these findings highlight how exercise modality influences gut microbial diversity, with HIFT promoting a more favorable microbial profile compared to traditional endurance training. Understanding these effects can help tailor exercise programs to improve both fitness and gut health.

**Supplementary Information:**

The online version contains supplementary material available at 10.1186/s13619-025-00244-y.

## Background

Physical activity has been shown to influence gut microbiota composition, which in turn can impact various aspects of health, including digestion, immunity, and metabolism. Despite the growing interest in understanding how different exercise modalities shape the gut microbiome, limited research has directly compared the microbial profiles of individuals engaged in moderate-intensity continuous training (MICT), high-intensity interval training (HIIT), and high-intensity functional training (HIFT). Traditional endurance training typically focused on longer-duration, moderate-intensity sessions without rest, which was often referred to as MICT and HIIT, denoting the alternating of short bursts of high-intensity exercise and recovery periods, has emerged as a prevalent alternative mainly due to its time efficiency. There is growing evidence indicating that HIIT might yield superior improvement in cardiorespiratory fitness than MICT in both healthy (Milanović et al. 2015) and chronic illness populations (Elliott et al. 2015; Liou et al. 2016). Nevertheless, an exercise modality that has gained great popularity in recent years is HIFT. HIFT is an exercise modality that emphasizes functional, multi-joint movements, which can be adapted to any fitness level and induce greater muscle recruitment compared to more traditional exercise (Feito et al. 2018).

The gut microbiota is a complex ecosystem that plays a crucial role in overall health, encompassing aspects such as digestion, immunity, and mental well-being. Previous evidence has emerged that exercise is associated with alterations in the gut microbiota (Allen et al. 2018; Hamasaki 2017). A recent study by Resende AS et al. revealed that moderate aerobic exercise intensity improved cardiorespiratory fitness and affected gut bacteria composition (Resende et al. 2021). Another study also indicated that compared with less physically active esports players, the bacterial diversity was increased in professional athletes (Kulecka et al. 2022). Although intriguing alterations in gut microbiota resulting from exercise interventions have been reported, the specific shifts in the gut microbiome among individuals participating in one of these three exercise modalities (MICT, HIIT, and HIFT) remain unclear.

This study aimed to fill this gap by utilizing 16S rRNA gene sequencing to examine the gut microbiota profiles of participants in MICT, HIIT, and HIFT groups.

## Results

### General characteristics of study participants

Prior to the intervention, body composition, autonomic nerve function, and cardiopulmonary endurance were assessed in the MICT group, HIIT group, and HIFT group. The results indicated that the pre-test values for each indicator showed no significant differences between the groups (*P* > 0.05), as shown in Table S1. In addition, as shown in Table S2, the results of the analysis of variance (ANOVA) indicated significant differences among the three groups in waist-to-hip ratio, visceral fat area, sympathovagal balance index (LF/HF ratio), peak oxygen uptake per kg, and heart rate at anaerobic threshold (all *P* values < 0.05). The multiple comparisons revealed that HIIT group exhibited a significantly higher heart rate at anaerobic threshold compared to the MICT group. In contrast, the HIFT group demonstrated more pronounced improvements than the MICT group across multiple parameters, including waist-to-hip ratio, visceral fat area, sympathovagal balance index, and heart rate at anaerobic threshold. Moreover, compared to HIIT group, the HIFT group showed significantly greater improvements in waist-to-hip ratio, visceral fat area and peak oxygen uptake per kg. These findings suggest that among the different exercise modalities, HIFT has a more pronounced impact on body composition, autonomic nervous function, and cardiopulmonary fitness.

### Profiles of gut microbiota among MICT, HIIT, and HIFT groups

The bacterial 16S rRNA from stool samples of participants were extracted and sequenced using the Illumina platform (Fig. S1). A total of 3,288,017 high quality sequences were generated, with an average of 106,065 sequences per sample. The total number of OTU identified in MICT group was 2,206, in the HIIT group was 3,812, and in the HIFT group was 3,960, which was higher in HIFT than that of MICT group (Fig. S2A). Meanwhile, Krona plots illustrated the relative abundance of bacterial communities at each taxonomic level—from phylum to genus—in fecal samples from MICT, HIIT, and HIFT subjects, based on the taxonomic assignments of 16S rRNA gene sequences (Fig. S2B-D).

**Fig. 1:**
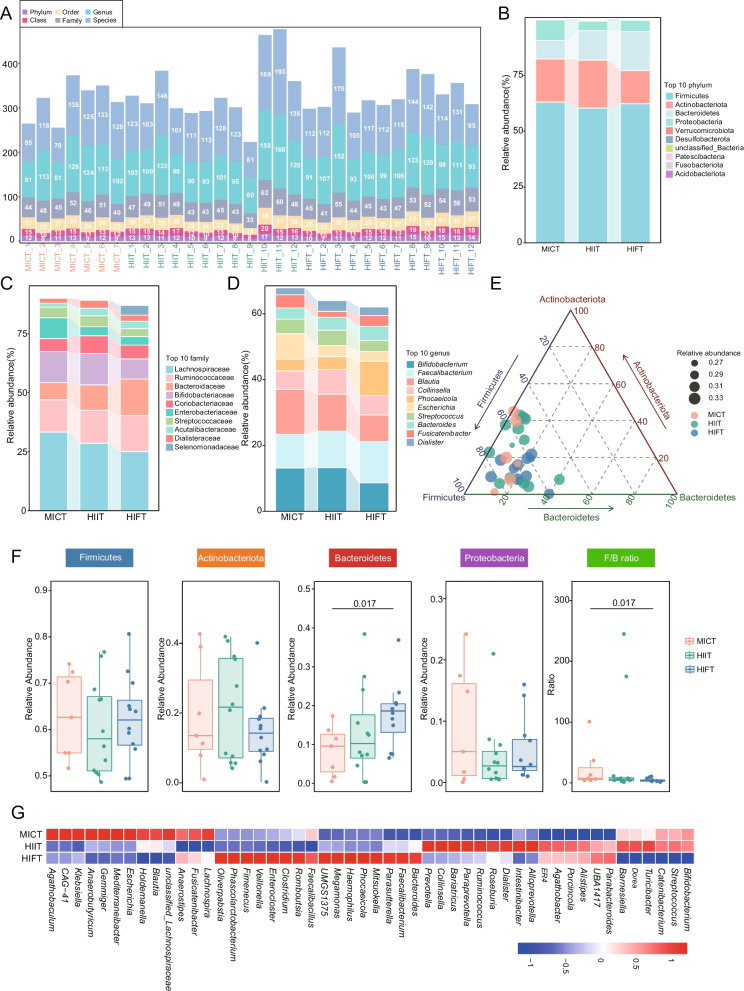
Profiles of gut microbiota among MICT, HIIT and HIFT groups. **A** The number of intestinal flora in phyla, classes, orders, families, genera and species identified in each participant in the MICT, HIIT and HIFT group. **B**-**D** The intestinal microbial composition of each group is depicted by the relative abundance of the most abundant phyla, families and genera (top 10). **E** The distribution of participants was plotted in a ternary diagram based on the relative abundance of the top 3 most dominant phyla (Actinobacteriota, Firmicutes, and Bacteroidetes). **F** Comparison was performed to assess the discrepancy in the top four phyla and the Firmicutes/Bacteroidetes (F/B) ratio between the groups. **G** Heatmap depicting the relative abundance and distribution of the predominant genera across different groups. Abundance profiles are standardized into Z scores by subtracting the mean abundance and dividing the standard deviation of all samples. A negative Z score, represent in blue, indicates that the row’s abundance is below the mean; while a positive Z score, shown in red, signifies an abundance above the mean. MICT, Moderate-intensity continuous training; HIIT, high-density interval training; HIFT, High-intensity functional training

The microbial analysis of the fecal samples from MICT, HIIT, and HIFT groups was conducting using 16S rRNA gene sequencing. The total number of identified bacterial phyla, classes, orders, families, genera, and species in each sample is presented in Fig. 1A. Based on these observations, the composition of the bacterial community was further analyzed in different groups at the phylum level (Fig. 1B), and the 10 most abundant bacterial families and genera were shown for individual samples within each group (Fig. 1C-D) and individual samples (Fig. S3). The distribution of participants was visualized in a ternary diagram based on the relative abundance of the top 3 most dominant phyla (Firmicutes, Actinobacteriota, and Bacteroidetes) (Fig. 1E). Notably, for the most abundant phyla in the groups, Bacteroidetes differed markedly among the samples of MICT, HIIT, and HIFT groups, showcasing a progressive increase from MICT to HIFT group. Additionally, the ratio of Firmicutes/Bacteroidetes showed a gradual reduction from the MICT to the HIFT group. Unfortunately, no significant differences were observed in the abundances of Firmicutes, Actinobacteriota, as well as Proteobacteria among the groups (Fig. 1F). The genera detected in the subjects also exhibited disparate abundance across MICT, HIIT and HIFT, with *Escherichia*, *Klebsiella* etc. reduced in HIIT and HIFT groups, while *Alistipes*, *Parabacteroides* etc. elevated in HIIT and HIFT groups (Fig. 1G).

### Gut microbial diversity among MICT, HIIT, and HIFT groups

**Fig. 2:**
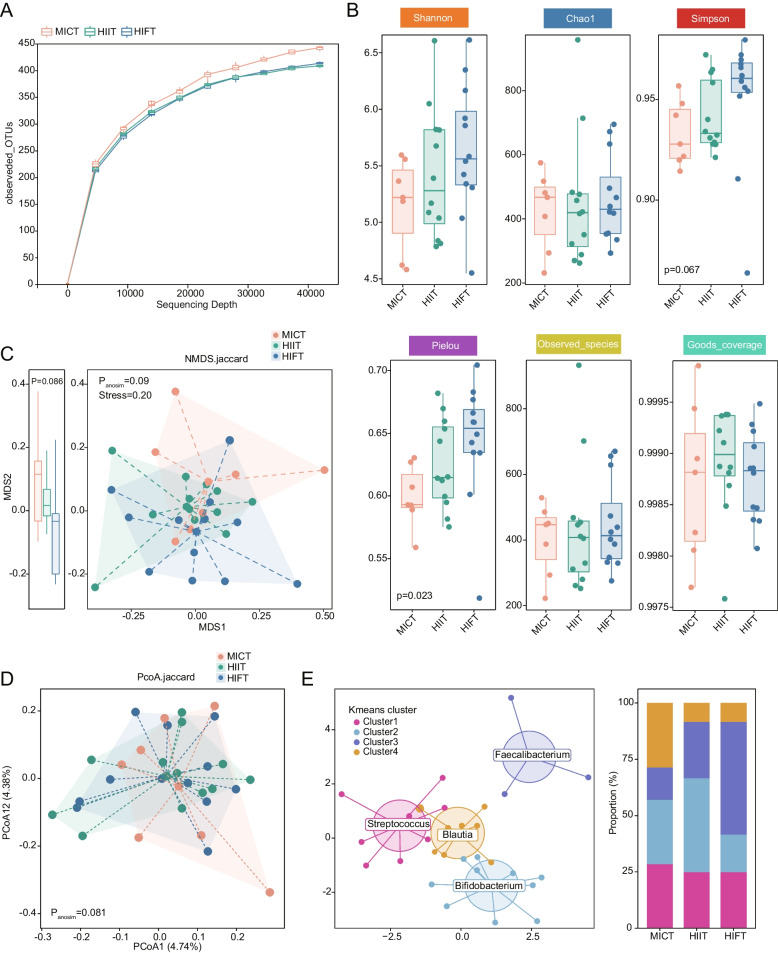
Characteristics of microbial alpha-diversity and beta-diversity among MICT, HIIT and HIFT. **A** The rarefaction curve elucidates the trend of variation in OTUs across samples with increasing sequencing depth. **B** Alpha-diversity (within-habitat) parameters are shown: Shannon diversity, Chao1 richness, Simpson’s index, Pielou evenness, Observed species, and Goods coverage. *P*-values are derived from the Kruskal–Wallis test. The boxes represent the interquartile ranges, and the lines inside indicate the medians. **C**-**D** Beta-diversity (between-habitat) of each group, including NMDS (**C**) and PCoA (**D**), was assessed based on Jaccard distance. Different colors in the scatter plots represent samples from distinct groups. The higher the similarity between samples, the closer they distribute in the plots. Axis 1 in the NMDS plot accounts for the variations among groups. *P*-values derived from ANOSIM analysis. Box plot depicts the distribution of single coordinate axis in MDS2. **E** All the participants were assigned into discrete clusters as identified by K-means clustering of genus-level features. The percentage of MICT, HIIT and HIFT samples distributed in Cluster1, Cluster2, Cluster3, and Cluster4. The top genera in each cluster are labeled, with *Streptococcus* prominent in cluster type1, *Bifidobacterium* dominant in cluster type2, *Faecalibacterium* in cluster type3, and *Blautia* prominent in cluster type4. There were 28.6% MICT, 25% HIIT and 25% HIFT in cluster type1; 28.6% MICT, 41.7% HIIT and 16.7% HIFT in cluster type2; 14.3% MICT, 25% HIIT and 50% HIFT in cluster type3; 28.5% MICT, 8.3% HIIT and 8.3% HIFT in cluster type4. NMDS, Nonmetric dimensional scaling; PCoA, Principal coordinate analysis

To investigate the variations in the intestinal bacterial community among MICT, HIIT, and HIFT groups, alpha- and beta-diversity analyses at the genus level were conducted based on the sequencing data. As shown in Fig. 2A, the flattened rarefaction curves indicated that the sequencing results reflected the diversity of the samples, suggesting that further increases in sequencing depth would not detect any additional OTUs. The alpha-diversity (Shannon index, Simpson index and Pielou evenness) indicated gradually increased from the MICT group to the HIFT group, especially the Pielou evenness (*P* = 0.023), which showed significant statistical differences among the three groups (Fig. 2B). Meanwhile, a slightly difference in beta-diversity was identified among the groups using NMDS (*P* = 0.09, Anosim analysis) and PCoA (*P* = 0.081, Anosim analysis) based on Jaccard distance (Fig. 2C-D). In addition, all participants were assigned into discrete clusters as identified by Kmeans clustering of genus-level features. The resulting Cluster 1, Cluster 2, Cluster 3, and Cluster 4 were predominantly characterized by the genera Streptococcus, Bifidobacterium, Faecalibacterium, and Blautia, respectively (Fig. 2E). The analysis revealed a progressively increase proportion of samples in Cluster 3 from MICT to HIIT and then to HIFT group, with 14.3% of MICT, 25.0% of HIIT group and 50.0% of HIFT group.

### Gut bacteria discriminate between MICT and HIIT, as well as between MICT and HIFT

To investigate the gut microbial compositions of participants in the study cohort, Mfuzz analysis was performed to assess the abundance changes of all genera as individuals progressed from MICT to HIIT, and subsequently to the HIFT group (Fig. S4A-H). Based on the distinct variation patterns of genera across different groups, eight disparate clusters were identified. For instance, Cluster 3 comprises a total of 32 genera, with the most enriched top 10 being *Fusicatenibacter*, *Anaerostipes*, *Faecalibacillus*, *Lachnospira*, etc. (Fig. S4C). These bacteria exhibited relatively lower abundance in the HIIT group and were elevated in MICT and HIFT groups. Conversely, there were 115 genera in Cluster 7, predominantly including *Collinsella*, *Prevotella*, *Bariatricus*, *Paraprevotella* etc., which exhibited reduced enrichment in HIIT group, other than those in the MICT and HIFT groups (Fig. S4G).

In addition, Cluster 5 contained 44 genera, including *Blautia*, *unclassified_Lachnospiraceae*, *Holdemanella*, *Catenibacterium*, whereas Cluster 6 included 47 genera, such as *Faecalibacterium*, *Bacteroides*, *Parabacteroides*, *Parasutterella*. In Cluster 5, gut microbial abundance was progressively reduced across groups, while in Cluster 6, it was progressively enhanced across groups (Fig. S4E-F). Subsequently, correlation analyses were conducted, revealing a significant positive correlation between the intestinal bacteria in Cluster 5 and Cluster 6 (Fig. S4I-J).

**Fig. 3:**
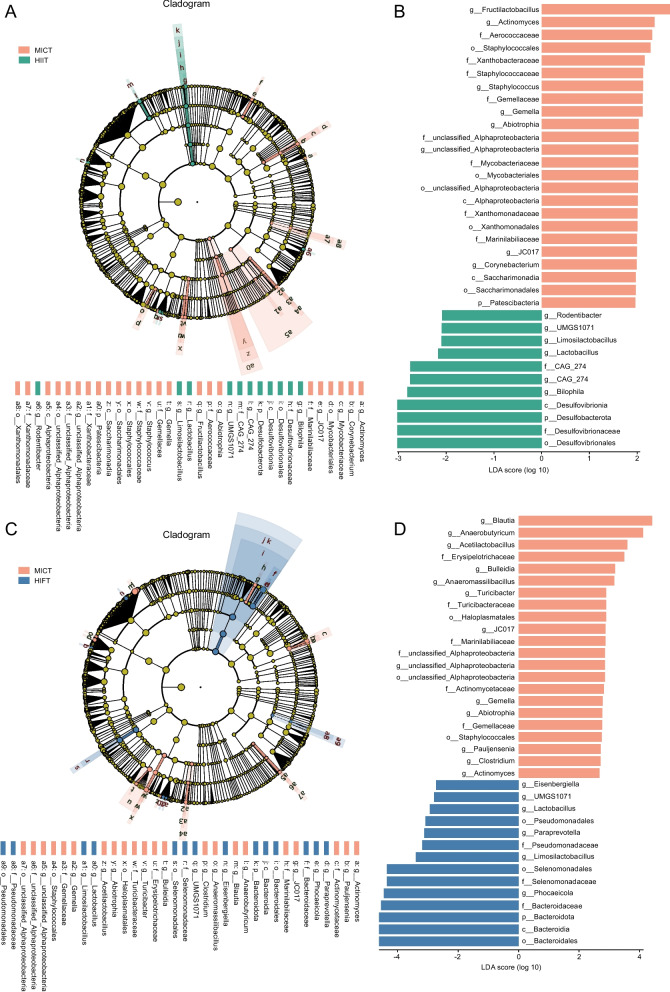
Gut bacteria discriminate between MICT and HIIT, as well as between MICT and HIFT. **A**-**B** The contrasting taxonomy composition between MICT and HIIT groups is elucidated through Linear discriminant analysis (LDA) effect size (LEfSe) analysis based on LDA score > 2 and *P* value < 0.05. Cladogram showed the taxonomic tree and enriched groups of distinct taxa, with differential abundant taxonomic clades at phylum, class, order, family and genus level shown by successive circles from the inner to outer rings. **C**-**D** LEfSe analysis assesses the differential taxonomic compositions between the MICT and HIFT groups. LDA, Linear discriminant analysis; LEfSe, LDA effect size analysis

To explore the gut microbial changes in HIIT and HIFT groups, differential abundant microbiota as compared with the MICT group were identified by LEfSe analysis (Fig. 3). As shown in Fig. 3A, a total of 35 discriminant taxa, of which 24 were enriched in the MICT group and 11 were abundant in HIIT group were detected, such as *Lactobacillus*, *Bilophila*, *Limosilactobacillus*, *Rodentibacter* in the HIIT group, contrasted with *Corynebacterium* and *Staphylococcus* in the MICT group. Meanwhile, in comparison with MICT group, 22 significant elevated and 14 markedly reduced bacteria were found in the HICT group (Fig. 3B). Remarkably, *Lactobacillus* and *Limosilactobacillus* were also confirmed to be flourished in the HIFT group, and we further discovered increased *Eisenbergiella*, *Paraprevotella* and depleted *Actinomyces*, *Anaeromassilibacillus* in the HIFT group. Furthermore, a correlation network analysis was performed between these core intestinal microbiome at the genus level and clinical characteristics (Fig. S5A). The differentially abundant core microbes identified between MICT and HIIT, as well as those between MICT and HIFT, were found to be associated with exercise training components and physical fitness parameters. For instance, the abundance of *Actinomyces* exhibited a negative correlation with peak oxygen intake per kg and peak oxygen intake. On the contrary, there was a positive correlation between *Anaeromassilibacillus* and body fat percentage, visceral fat area and waist-to-hip ratio. Moreover, the association between core differential KEGG pathway, enzymes, and exercise training parameters was also assessed. Notably, we identified that EC: 3.4.11.14 (alanine aminopeptidase) exhibited a positive correlation with peak oxygen intake per kg and anaerobic threshold oxygen uptake per kg, while demonstrating a negative correlation with waist-to-hip ratio and LF/HF ratio (Fig. S5B).

### KEGG pathways and enzymes summarized the alterations in gut microbial functions in individuals undergoing MICT, HIIT, and HIFT

**Fig. 4:**
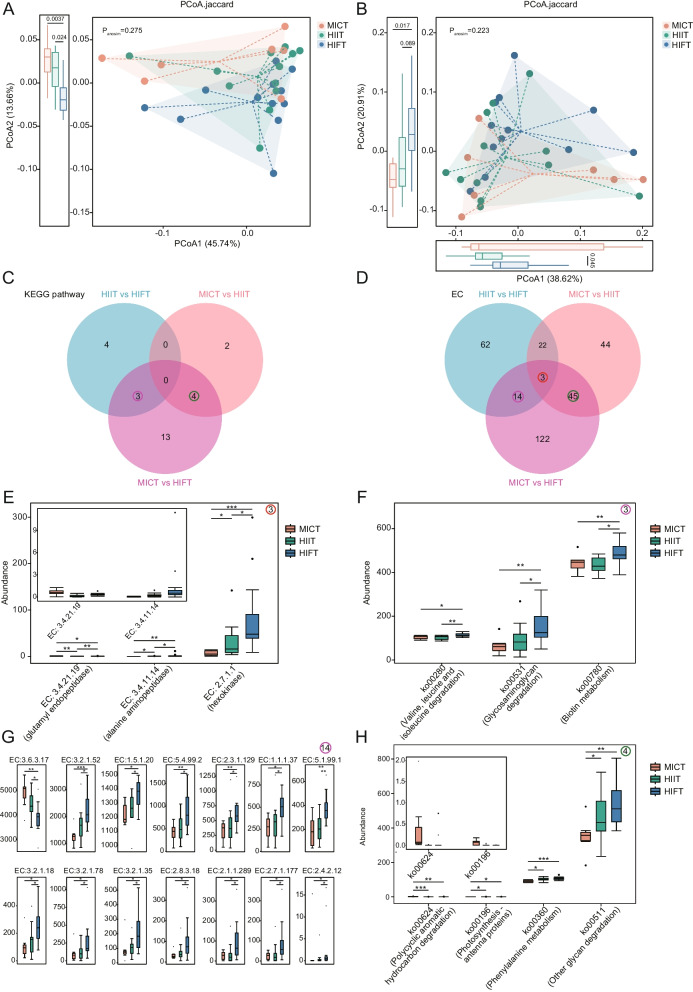
Gut microbial functions alterations in individuals undergoing MICT, HIIT and HIFT were summarized in KEGG pathways and enzymes. **A**-**B** Scatter plots from PCoA analysis based on Jaccard distance are generated to illustrate the predicted KEGG pathway (**A**) and enzyme (EC) profiles (**B**) in the samples analyzed by PICRUST2. Different colors in the scatter plots represent samples from distinct groups. The higher the similarity between samples, the closer they distribute in the plots. *P* values are obtained from the Anosim test to evaluate the significance of heterogeneity among individuals within MICT, HIIT and HIFT groups. Box plots describe the distribution of groups in PCoA1 or 2, with differences determined by wilcoxon rank sum test. **C**-**D** Venn diagrams of differential microbial KEGG pathways (**C**) and ECs (**D**) altered in comparisons of MICT vs. HIIT, MICT vs. HIFT and HIIT vs. HIFT, respectively. **E** Box plots show the common ECs simultaneously varied among distinct comparisons. **F** Box plots depict the shared KEGG pathways that exhibited simultaneous variation across the comparisons of HIIT vs. HIFT and MICT vs. HIFT. **G** The 14 ECs were specifically identified as altered in the HIFT group when compared with MICT and HIIT. **H** The four KEGG pathways shifted in the MICT group when compared to both the HIIT and HIFT groups. *P < 0.05, **P < 0.01 and ***P < 0.001

According to the KEGG database, we conducted an elevation of gut microbial functions across different groups, annotating a total 170 KEGG pathways and 2065 enzymes. No obvious separation was seen in PCoA based on KEGG pathways (*P* = 0.275, Anosim analysis, Fig. 4A) and ECs (*P* = 0.223, Anosim analysis, Fig. 4B) among the groups. Yet, the distribution of samples along the second PCoA for KEGG pathway revealed significant differences when comparing HIFT with MICT and HIIT (*P*_PCoA2 (MICT vs. HIFT)_ = 0.0037, *P*_PCoA2 (HIIT vs. HIFT)_ = 0.024, Fig. 4A). Additionally, the first PCoA for enzymes indicated a value of *P*_PCoA1 (HIIT vs. HIFT)_ = 0.045, while the second PCoA for enzymes showed *P*_PCoA2 (MICT vs. HIFT)_ = 0.017, Fig. 4B). These findings suggest that there are notable differences in KEGG pathways and enzyme distributions to some extent among these exercise modalities. A total of 26 KEGG pathways and 312 enzymes exhibited disparities across groups, as elucidated by the Wilcoxon rank sum test (Fig. 4C-D). It is worth noting that 3 enzymes (EC: 3.4.21.19, EC: 3.4.11.14 and EC: 2.7.1.1) were found to be synchronously alterations when comparative analyses were conducted between groups. The relative abundances of the differential enzymes shared in this comparison are illustrated in Fig. 4E. Intriguingly, the relative abundance of 2 out of these 3 enzymes—specifically, alanine aminopeptidase and hexokinase—exhibited a remarkable asymptotic elevation as progressed from MICT to HIIT, with further enhancement observed within HIFT group. At the same time, we discerned 3 KEGG pathways (ko00280, ko00531 and ko00780) and 14 enzymes that were significantly altered in then HIFT group when juxtaposed with both MICT and HIIT groups, as illustrated in Fig. 4F-G. The microbial capacities associated with valine leucine and isoleucine degradation (ko00280), glycosaminoglycan degradation (ko00531) and biotin metabolism (ko00780) significant enhancement within the HIFT group. A plethora of enzymes, such as EC:3.2.1.52, EC:1.5.1.20, EC:5.4.99.2, and EC:2.3.1.129, etc. were found to be increased dramatically in HIFT group, while EC:3.6.3.17 were diminished. In addition, we also identified another 4 KEGG pathways and 45 enzymes significantly altered in the MICT group compared to the HIIT and HIFT groups, as demonstrated in Fig. 4H and Fig. S6.

**Fig. 5:**
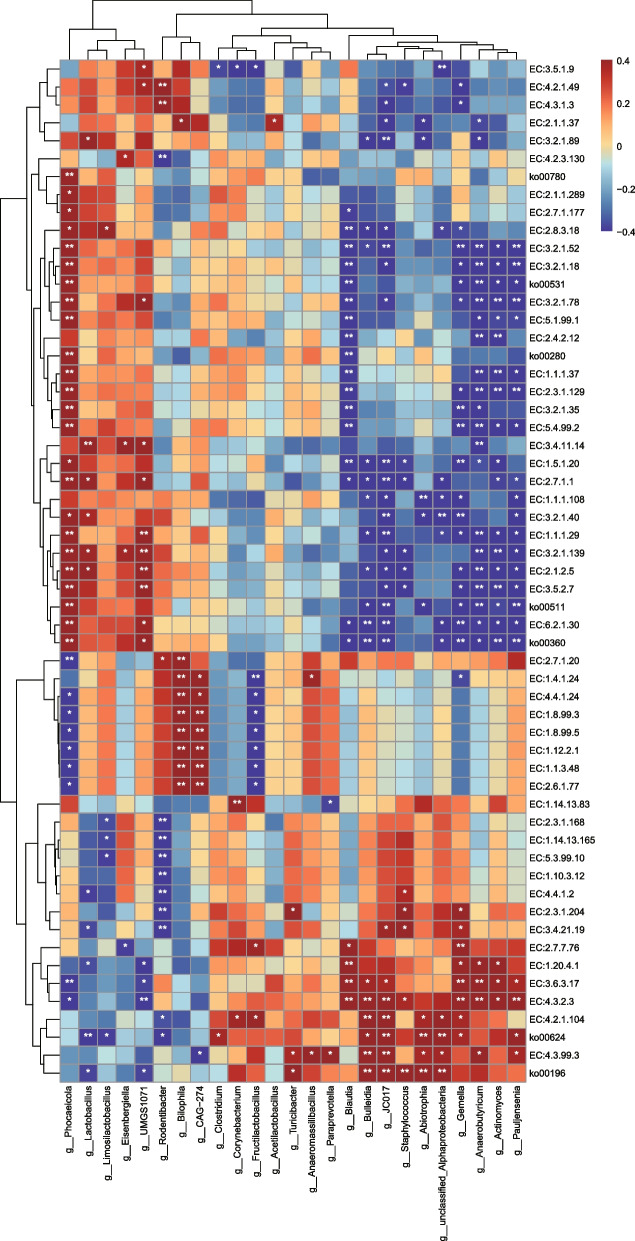
Correlation of the microbial core genera, and differential KOs, ECs. The correlation between the key differential genera listed in Fig. 3 and the core KOs/ECs (identified in Fig. 4) is determined with Spearman’s correlation analysis. The heatmap describes the positive (red) and negative (blue) associations based on |r|. The statistical significance is labeled with *P < 0.05 and **P < 0.01, respectively

Furthermore, a Spearman’s correlation analysis was conducted to examine between the significant distinct gut microbial functions and discriminant genera (Fig. 5). It was suggested that genera abundant in the MICT group, such as *Actinomyces*, *Anaerobutyricum*, are positively correlated with a majority of enzymes, including EC:1.20.4.1, EC:4.3.2.3, EC:3.6.3.17, etc.; while these genera are also negatively correlated with certain other KEGG pathways (ko00360, ko00511, ko00531) and enzymes (EC:1.5.1.20, EC:5.4.99.2 and EC:3.2.1.52). On the contrary, the core gut microbes showing differential enrichment in HIIT and HIFT compared to MICT group (*Phocaeicola*, *Lactobacillus*, *Eisenbergiella*) were found to be positively correlated with EC:3.4.11.14 and ko00360, while demonstrating negatively correlations with EC:2.7.7.76 and EC:4.3.2.3.

## Discussion

In the current study, a thorough analysis was undertaken using 16S rRNA gene sequencing to explore the altered microbial community in MICT, HIIT, and HIFT groups. Our findings revealed distinct gut microbiome profiles in participants under different exercise modes. Notably, the alpha-diversity gradually increased from the MICT group to the HIFT group. Slight variations in beta-diversity were observed among the different groups. Furthermore, there was a progressive shift towards a Faecalibacterium-dominated microbial type from HIIT to HIFT group compared to MICT group. More importantly, individuals in the HIFT group were identified to be enriched with *Lactobacillus* and *Limosilactobacillus*, along with reduced *Actinomyces* and *Anaeromassilibacillus*. The above-mentioned intestinal microbiota, along with the differential KEGG pathways and enzymes, are related to exercise training components and physical fitness parameters.

Bacterial diversity is recognized as a characteristic associated with health status and various diseases (Wang et al. 2021). We demonstrated that HIIT and HIFT exercise leads to effective improvements in gut microbiota diversity as evidenced by higher α-diversity and an inclination to Faecalibacterium-dominated type. These findings corroborate previous works that individuals with higher physical activity or fitness levels exhibited a higher diversity in gut microbiome and a greater variety in health-promoting bacteria (Forsythe et al. 2008). Furthermore, we also discovered that the ratio of Firmicutes/Bacteroidetes exhibited a gradual reduction from the MICT to the HIFT group. As is well established, bacteria from the phyla Firmicutes and Bacteroidetes are the most predominant, representing 90% of the gut microbiota (Stojanov 2020). An increased Firmicutes/Bacteroidetes ratio is associated with obesity; specifically, this ratio is increased in obese mice compared to their lean counterparts (Ley et al. 2005). Among adult populations, body mass index has been positively correlated with the Firmicutes/Bacteroidetes ratio (Koliada et al. 2017). Additionally, strength exercise, a form of resistance training, has been shown to reduce the ratio of Firmicutes/Bacteroidetes, which was in line with our findings (Chen et al. 2021).

Moreover, by characterizing the variations in the taxonomic composition of the bacterial community, a slight difference in beta-diversity was identified among the MICT, HIIT and HIFT. Specifically, certain bacteria, such as *Lactobacillus*, *Limosilactobacillus*, and *Eisenbergiella* were prominently enriched in the HIIT or HIFT group; whereas *Corynebacterium* and *Staphylococcus* were predominant in the MICT group. *Lactobacillus* is a well-known probiotic and has been reported to exert numerous health benefits (Reid et al. 2011). Indeed, a recent study indicated that supplementation of *Lactobacillus* in rats after myocardial infarction elicited cardioprotective effects (Gan et al. 2014). Moreover, *Lactobacillus* supplements notably enhanced muscle mass, strength, and endurance capacity, which can restore age-related muscle loss (Liu et al. 2021). Sugimura Y et al. indicated that *Eisenbergiella* is positively associated with appendicular skeletal muscle/body weight, might help increase skeletal muscle mass (Sugimura et al. 2022). It is well-known that HIFT is a training paradigm derived from both HIIT and strength exercise, capable of eliciting greater muscle recruitment than repetitive aerobic exercises (such as MICT) (Ben-Zeev and Okun 2021). To some extent, this is the reason why *Lactobacillus* is enriched in the HIIT and HIFT groups in compared with the MICT group.

Functionally, the KEGG pathway and enzyme analysis predicted based on PICRUST2 disclosed that the abundance of ko00280 (valine, leucine, and isoleucine degradation), ko00531 (glycosaminoglycan degradation) and EC: 3.4.11.14 (alanine aminopeptidase) was elevated in the HIFT group. Simultaneously, it was revealed that both *Lactobacillus* and *Eisenbergiella* were positively correlated with EC: 3.4.11.14 (alanine aminopeptidase, the EC changed synchronously in comparative analysis between groups). To our knowledge, the proliferative capacity of myoblasts is of crucial significance for skeletal muscle formation. Remarkably, a previous study has indicated that the activities of proteasomes and aminopeptidases in the proteolysis pathway are indispensable for myoblast proliferation and differentiation. The reduction of alanine aminopeptidase enzymatic activity impaired the cell cycle progression in C2C12 myoblasts (Osana et al. 2020). Interestingly, in the present study, the relative abundance of alanine aminopeptidase was the highest in the HIFT, potentially related to the enhanced muscle recruitment observed in this training paradigm. This finding further substantiates the hypothesis that the bacterial communities in the HIFT group might contribute to the beneficial effects of this training method on muscle mass. Valine, leucine, and isoleucine, as the branched chain amino acids, exert critical roles in the regulation of energy homeostasis, nutrition metabolism, immunity, and disease in both human beings and animals (Nie et al. 2018). A previous study demonstrated that basal protein degradation is indispensable for ensuring optimal skeletal muscle function (Stokes et al. 2018). This finding aligns with our research results, which reveal that the degradation of valine, leucine, and isoleucine, is particularly prominent during HIFT, thereby guaranteeing that muscles maintain a high level of expression as a consequence of intense exercise during HIFT.

Nevertheless, this study also has several limitations. Firstly, being a single-center study, it is of utmost urgency to perform extensive researches involving multiple centers in order to replicate and further validate the alterations in profiles of microbiota among individuals under different exercise modalities. Secondly, the sample size is relatively small. Here, rarefaction curves were conducted to assess the sufficiency of sample in each group. It was found that the curves tended to be flat, indicating that there would be a handful of new species characteristics yielded when more samples were included. Thirdly, although 16S rRNA sequencing is suitable for high-throughput profiling of gut microbiota, it could not achieve the same resolution as whole-genome sequencing. 16S rRNA sequencing often struggles to differentiate between closely related species, resulting in an incomplete understanding of the microbial community structure at the species levels. Moreover, this offers limited insights into the functional capabilities of the microbiome, which could potentially clarify the more detailed mechanisms underlying the association between exercise modality and gut microbiota shifts. Therefore, further research utilizing more advanced sequencing technologies is needed to gain a deeper understanding of the microbial ecosystem and its functional implications among these participants. Lastly, diet and medication are critical modulators of the gut microbiome. While, in our study, all participants were healthy university students living on campus and consuming meals primarily from centralized dining facilities. This standardized living environment likely reduced inter-individual variability in dietary habits, providing a natural control of dietary influences on the gut microbiome. Furthermore, stringent inclusion criteria that participants were free from chronic diseases and not taking regular medications at the time of sampling, minimizing medication-related confounding effects. In the future, prospective studies incorporating longitudinal dietary monitoring and medication tracking would be valuable to further validate our findings and disentangle these complex interactions.

## Conclusions

In summary, these data indicate for the first time that different exercise paradigms (MICT, HIIT, and HIFT) exert differential effects on gut microbiome composition and function using 16S rRNA gene sequencing. Compared with MICT, individuals undertaking HIIT and especially HIFT exercise resulted in increased alpha-diversity, an inclination to Faecalibacterium-dominated type, along with enriched *Lactobacillus*, *Limosilactobacillus*, and *Eisenbergiella*. Furthermore, the specific KEGG pathway (valine, leucine, and isoleucine degradation) and the enzyme (alanine aminopeptidase), which might be related to muscle function, were predominant in HIFT.

## Methods

### Study cohort and sample collection

A total of 31 untrained, healthy university students voluntarily participated in this study. Inclusion criteria stipulated that participants must have a body mass index (BMI) within the range of 18 and 24 kg/m^2^. According to the International Physical Activity Questionnaire (IPAQ), all participants were classified as not engaging in regular high-intensity physical activity and reported a total weekly physical activity level of less than 600 MET-min/week. Individuals with chronic diseases (such as cardiovascular disease, metabolic disorders, or cancer), mental health conditions (e.g., depression, anxiety, or obsessive–compulsive disorder), physical injuries or movement restrictions (e.g., musculoskeletal disorders) were excluded. Additionally, participants were also excluded if they had received antibiotics or probiotics within the past two months.

The study utilized a randomized controlled trial design, with participants randomly assigned to one of three groups: a stair-climber-based high-intensity interval training (HIIT) group (*n* = 12), a high-intensity functional training (HIFT) group (*n* = 12), or a moderate-intensity continuous running (MICT) group (*n* = 7). Throughout the intervention, participants maintained their habitual dietary intake and lifestyle patterns, including sleep, sedentary behavior, and physical activity. The use of nutritional supplements and engagement in vigorous physical activity beyond their usual routines were strictly prohibited during the intervention.

Prior to the commencement of the study, all participants were thoroughly informed about the experimental procedures and provided written informed consent. Ethical approval for the study was obtained from the Biomedical Research Ethics Committee of the Capital University of Physical Education and Sports (approval number: 2021A27).

### Exercise intervention program

A one-week pre-intervention familiarization period was conducted to ensure participants were accustomed to the training protocols. The HIIT, HIFT, and MICT interventions were performed three times per week—on Mondays, Wednesdays, and Saturdays—for a total duration of eight weeks. If a participant was unable to attend a scheduled session, the session was rescheduled for the following day and supervised by the same researcher. All training sessions across the three groups began with a standardized 5-min warm-up consisting of low-to-moderate intensity running and stretching, followed by the main training session, and concluded with a 5-min cool-down, including relaxation and stretching.

#### HIIT group

Participants in the HIIT group followed a cycling protocol using a cycle ergometer. After a 10-min warm-up, participants performed 10 high-intensity intervals, each consisting of 45 s of cycling at near-maximal intensity, followed by 90 s of active recovery. The resistance level on the ergometer was individually calibrated before the intervention to ensure an appropriate workload. During the sprint intervals, participants worked at 80%−85% of their maximum heart rate (HRmax), while recovery periods were maintained at 40%−45% HRmax. The session concluded with a 5-min recovery phase. Key performance metrics, including resistance, pedaling cadence (50~70 rpm), heart rate (monitored using Polar H9 devices, recorded in beats per minute), and power output (measured in watts), were continuously monitored to ensure participants achieved the target intensity.

#### HIFT group

The HIFT group underwent an intervention based on CrossFit (CF) principles. The specific protocol was adapted from the Whole-Body Aerobic Resistance Training Circuit Group (WBARCG) program introduced by Terrence and Matthew. Participants performed a sequence of functional bodyweight exercises, completing 7~9 movements per session (Table S1). The goal was to complete as many circuits of the exercise sequence as possible within 30 min. No rest periods were permitted between exercises or circuits. Each session lasted 30 min, with the training frequency matching that of the HIIT group. All HIFT sessions were video-recorded, and participants were given access to the instructional video prior to the intervention to ensure familiarity with the movements and procedures. During the intervention, the video was displayed on a screen to help participants maintain the proper pace and rhythm for each exercise.

#### MICT group

Participants in the MICT group performed treadmill-based running exercises. The intensity of the exercise was maintained at 60%~70% of HRmax, calculated as 220 minus the participant's age. Each session lasted 40 min and included three phases: a 5-min walking warm-up, 30 min of continuous running, and a 5-min walking cool-down. To ensure the prescribed intensity was achieved, participants’ heart rates were continuously monitored throughout the session using an activity wristband. The peak heart rate (HRpeak) for each session was required to reach at least 75% of HRmax, as determined by Cooper’s 12-min running test.

### Data collection

Body composition was assessed using the InBody760 BIA analyzer (software v120.4.0.0.7) under standard conditions (morning fast, 22~24℃), employing eight-point tactile electrodes (accuracy error < 1.5%) to measure body mass index (BMI), body fat percentage, waist-to-hip ratio, and visceral fat area. Autonomic nervous system function was evaluated via the Fourier FLY-2 Physiological Monitor. Participants were instructed to abstain from strenuous exercise for 48 h and avoid caffeine and alcohol consumption for 12 h prior to testing. Peripheral vascular pulsation signals were acquired via photoplethysmography (PPG) and subsequently analyzed using the ANS-1 Analysis System (V6.6, Beijing Fourier) to derive time-domain (such as mean RR interval, standard deviation of normal-to-normal intervals (SDNN)) and frequency-domain parameters (such as Low Frequency to High Frequency ratio, (LF/HF ratio)). Cardiopulmonary exercise testing (CPET) was conducted based on the modified Bruce protocol, utilizing a symptom-limited exercise test. The test was performed on a Schiller Rehab-B ergometer bicycle with a stepwise incremental workload. Male participants underwent the test with a workload increment of 25 W/min, while female participants had an increment of 15 W/min, aiming to reach their maximum tolerable intensity within 8 to 12 min. Continuous physiological monitoring included 12-lead electrocardiogram (ECG) for heart rate assessment and breath-by-breath gas analysis for oxygen uptake via the PowerCube-Ergo metabolism system (LF8 V8.5 M SR4) with 20-s sampling intervals. Test termination required meeting ≥ 3 criteria: (1) respiratory exchange ratio (RER) > 1.05; (2) achievement of ≥ 90% ± 10% of the age-predicted maximum heart rate (calculated as 210—0.65 × age); (3) Borg rating of perceived exertion (RPE) ≥ 18; (4) oxygen uptake plateau (oxygen uptake increase of < 2.1 mL·kg⁻^1^·min⁻^1^ between adjacent workloads).

### Stool sample collection and 16S rRNA sequencing

Stool samples from each participant were freshly collected into containers placed on ice packs 48 h post sports. These samples were subsequently transported to the laboratory and stored at −80 °C until further processing. Total genomic DNA was extracted from the fecal samples, and the quality of DNA was evaluated by 1.2% agarose gel electrophoresis, with quantification being carried out using a Nanodrop spectrophotometer. A barcode sequence was incorporated, and the variable region of the rRNA gene was amplified through PCR. Briefly, each sample was diluted to 20 ng/μl, and the PCR reaction was set up with the following components: 5 μl of 5 × reaction buffer, 5 μl of 5 × GC buffer, 2 μl of dNTP (2.5 mM), 1 μl of Forward primer (10 μM), 1 μl of Reverse primer (10 μM), 2 μl of DNA Template, 8.75 μl of ddH2O, and 0.25 μl of Q5 DNA Polymerase. The primers utilized were as follows: forward primer 338 F (5’-ACTCCTACGGGAGGCAGCA-3’) and reverse primer 806R (5’-GGACTACHVGGGTWTCTAAT-3’). The detailed condition of amplified reaction was at initial denaturation 98 °C 2 min, denaturation 98 °C 15 s, annealing 55 °C 30 s, extension 72 °C 30 s, final extension 72 °C 5 min, 10 °C hold, and 25–30 Cycles. Quantification of the amplified products was accomplished with the Quant-iT PicoGreen dsDNA Assay Kit on a Microplate reader (BioTek, FLx800). The library was prepared with TruSeq Nano DNA Low Throughput Library Prep Kit, and purified using standard agarose gel electrophoresis. On Agilent Bioanalyzer, the library was assessed with Agilent High Sensitivity DNA Kit, and quantified with Quant-iT PicoGreen dsDNA Assay Kit on Promega QuantiFluor system, denatured to a single strand, and carried out using Illumina Novaseq-PE 250 platform.

The raw high-throughput sequencing data was processed using the Quantitative Insights Into Microbial Ecology (QIIME2 software), version 2019.4. Subsequently, the DADA2 algorithm was utilized for quality control, trimming, denoising, and assembly of the raw sequences to remove phiX, chimeric, and erroneous reads. Under Greengenes database (Release 13.8, http://greengenes.secondgenome.com/comments), the sequencing results were annotated with classify-sklearn methods (https://github.com/QIIME2/q2-feature-classifier).

### Microbial diversity

To assess the alpha diversity of the microbial community, richness was characterized by Chao1 and observed species indices; diversity was measured using the Shannon and Simpson indices; while evenness was represented by Pielou’s evenness index, and Good’s coverage index was used to depict coverage. Kruskal–Wallis rank sum tests were applied to conduct comparison of alpha diversity indexes among groups. Rarefaction curves were generated by extracting a certain number of sequences from each sample, and calculating the total number of operational taxonomic units (OTUs) that the sample contains and the relative abundance of each OTU in a given series of sequencing depths with the QIIME2 software. Curves exhibiting a flattening trend suggest that the sequencing data amount is progressively increasing and reasonable, with additional data contributing to the identification of a limited number of novel OTUs.

Beta diversity represents a comparative analysis of the microbial community composition regarding between-habitat diversity. Using the QIIME2 software, the beta diversity was evaluated by computing the Jaccard distance based on the abundance of OTU within each sample. Anosim analyses were conducted to determine the statistical significance of the differences among MICT, HIIT, and HIFT groups. Nonmetric dimensional scaling (NMDS) and principal coordinate analysis (PCoA) based on Jaccard distance were performed using vegan and the ggplot2 package in R.

### Functional annotation

The functional potential of the gut microbial community was predicted through the application of the phylogenetic investigation of communities by reconstruction of unobserved states (PICRUSt2) for generating the Kyoto Encyclopedia of Genes and Genomes (KEGG, https://www.kegg.jp/) ontology (KO) and enzyme profiles. By aligning 16S rRNA gene data from OTUs with the microbial reference genome Greengene database, a phylogenetic tree was established, and the gene function of their shared ancestor was inferred. Based on the copy number of the gene family corresponding to the reference sequence in the phylogenetic tree, the species having the closest sequence to the feature sequence was deduced, and thus the copy number of its gene family was obtained. The abundance of each sample’s characteristic sequence was used to calculate the copy number of the gene family. The metabolic pathway abundance data for each sample was obtained by mapping the gene families to the KEGG database and retrieving the corresponding pathway information. The bacterial community composition was mapped to the database, enabling the prediction of gene functions of gut bacteria.

### Statistical analysis

Quantitative variables with normal distributions are expressed as mean ± standard deviations (SDs), while quantitative data with nonnormal distributions are presented as median and interquartile range, and one-way ANOVA or Kruskal–Wallis rank sum tests were conducted for comparisons. Qualitative data are presented as number and percentage, and χ^2^ test was used for comparisons. Kmeans cluster analysis was conducted using the OmicStudio tools at https://www.omicstudio.cn/tool (Lyu et al. 2023). Unique bacterial taxa driving the specific group-specific differences were determined using the biomarker discovery algorithm linear discriminant analysis (LDA) effect size analysis (LEfSe) with parameters set at an LDA score of > 2.0 and *P* value < 0.05. In addition, differential genera with differential KO, EC, or clinical factors were estimated by Spearman’s correlation analysis. All statistical tests were two sided, and *P* less than 0.05 was considered as significant.

## Supplementary Information

Supplementary Material 1. Figure S1 An overview of the study design and participant characteristics. Figure S2 Overview for the gut microbes constitution among individuals engaged in MICT, HIIT and HIFT based on taxonomic levels. Figure S3 Distribution of bacteria at the phylum, family, and genus levels in the fecal microbiota of the current cohort comprising subjects engaged in MICT, HIIT and HIFT. Figure S4 Diverse fluctuation trend patterns of intestinal microbial general associated with various exercise modes, including MICT, HIIT and HIFT. Figure S5 Correlation of the microbial core genera KEGG pathways and enzymes and clinical parameters. Figure S6 The core enzymes specifically shift in MICT group.

Supplementary Material 2. Table S1. Test of homogeneity of all indicators before the intervention. Table S2. General characteristics of study participants. Table S3. Details of the functional high-intensity interval training intervention.

## Data Availability

The data sets supporting the results of this study have been deposited in the National Center for Biotechnology Information under BioProject accession code PRJNA1269258 (https://www.ncbi.nlm.nih.gov/sra/PRJNA1269258).

## Abbreviations

BMI: Body mass index
MICT: Moderate-intensity continuous training
HIIT: High-intensity interval training
HIFT: High-intensity functional training
HRmax: Maximum heart rate
HRpeak: Peak heart rate
LF: Low frequency
HF: High frequency
KEGG: Kyoto Encyclopedia of Genes and Genomes
KO: KEGG Ontology
EC: Enzyme
OTU: Operational taxonomic unit
PCoA: Principal coordinate analysis
NMDS: Non-metric multidimensional scaling
LDA: Linear discriminant analysis
LefSe: Linear discriminant analysis effect size
sd_HR: Standard deviation of heart rate
mean_RR: Mean RR intervals duration
SDNN: Standard deviation of normal-to-normal intervals
