# Supplementary material for: Impact of exercise-induced alterations on gut microbiota diversity and composition: comparing effects of different training modalities
Source: Cell Regen. 2025 Jul 2;14:28. doi: 10.1186/s13619-025-00244-y (PMC12222581; doi:10.1186/s13619-025-00244-y)
Supplement: Supplementary file 1 — Supplementary Material 1. Figure S1 An overview of the study design and participant characteristics. Figure S2 Overview for the gut microbes constitution among individuals engaged in MICT, HIIT and HIFT based on taxonomic levels. Figure S3 Distribution of bacteria at the phylum, family, and genus levels in the fecal microbiota of the current cohort comprising subjects engaged in MICT, HIIT and HIFT. Figure S4 Diverse fluctuation trend patterns of intestinal microbial general associated with various exercise modes, including MICT, HIIT and HIFT. Figure S5 Correlation of the microbial core genera KEGG pathways and enzymes and clinical parameters. Figure S6 The core enzymes specifically shift in MICT group. [file 13619_2025_244_MOESM1_ESM.zip › Figure Legends.docx]

**Figure S1 An overview of the study design and participant characteristics.** A total of 31 individuals are enrolled in the study, which involved random assignment to three groups: MICT group (n = 7), HIIT group (n = 12) and HIFT group (n = 12). The inclusion and exclusion criteria are comprehensively outlined in the Methods section. Gut microbiome composition is analyzed through 16S rRNA gene sequencing of stool samples. Data analysis encompassed an evaluation of microbial diversity, clusters, composition and function. Key findings include taxonomic distributions at phylum and genus levels, diversity metrics assessed via Pielou’s evenness indices, as well as microbial markers identified using LEfSe and LDA. Correlations between gut microbiome profiles and core pathways along with enzymes were examined using Spearman’s correlation.

**Figure S2 Overview for the gut microbes constitution among individuals engaged in MICT, HIIT and HIFT based on taxonomic levels.** (A) Venn diagram illustrating the quantity and proportion of shared and unique OTUs identified among the MICT, HIIT, and HIFT groups. The Overlapping regions represent the OTUs that are common to distinct groups. (B-D) The interactive presentations of taxonomic composition across MICT, HIIT and HIF groups are depicted using Krona charts, where circles represent the hierarchical taxonomic levels from phylum to genus, arranged from the innermost to the outermost layer. The size of each fan indicates the relative abundance of various taxa, with distinct colors assigned to differentiate between taxonomic units.

**Figure S3 Distribution of bacteria at the phylum, family, and genus levels in the fecal microbiota of the current cohort comprising subjects engaged in MICT, HIIT and HIFT.** (A) Taxonomic classification at the phylum level for each individual within the study cohort. (B-C) The relative abundances of top 10 most prevalent gut families and genera are presented for each individual across MICT, HIIT and HIFT groups.

**Figure S4 Diverse fluctuation trend patterns of intestinal microbial genera associated with various exercise modes, including MICT, HIIT and HIFT.** (A-H) The line chart of genera was derived from Mfuzz analysis, revealing eight clusters displayed distinct enrichment patterns from MICT, HIIT and HIFT. The total number of genera within each cluster is labeled. Abundance changes indicate the normalized level. The most abundant genus-level composition (top 10) of each Cluster is depicted in bar plot, respectively. (I) The network exhibiting the correlations of gut microbiota identified in Cluster 5 (identified in Figure S4E). (J) The network depicting the correlations of gut microbiota identified in Cluster 6 (identified in Figure S4F). Spearman’s correlation, P <0.05 and absolute coefficient >0.6. Positive correlations between nodes are represented by red connecting lines. Degree indicates the number of other nodes that each node was connected.

**Figure S5 Correlation of the microbial core genera KEGG pathways and enzymes and clinical parameters.** (A) Spearman’s correlation between altered gut genera and clinical parameters. The red color indicates positive associations between the clinical characteristics and bacteria taxa; blue color denotes negative associations. The statistical significance is labeled with *P < 0.05 and **P < 0.01, respectively. (B) Spearman’s correlation between altered KEGG pathways, enzymes and clinical parameters. The red color indicates positive associations between clinical characteristics and KEGG pathways or enzymes; blue color denotes negative associations. The statistical significance is labeled with *P < 0.05 and **P < 0.01, respectively. Mean_RR, mean RR intervals duration; SDNN, standard deviation of normal-to-normal intervals; BMI, body mass index; LF, low frequency; HF, high frequency.

**Figure S6 The core enzymes specifically shift in MICT group.** (A) The relative abundance of the top 16 of 45 enzymes and their distribution in the MICT, HIIT and HIFT groups. Wilcoxon rank-sum tests (*P < 0.05, **P < 0.01, ***P < 0.001).
