# Supplementary figures and images for: Impact of exercise-induced alterations on gut microbiota diversity and composition: comparing effects of different training modalities
Source: Cell Regen. 2025 Jul 2;14:28. doi: 10.1186/s13619-025-00244-y (PMC12222581; doi:10.1186/s13619-025-00244-y)

A

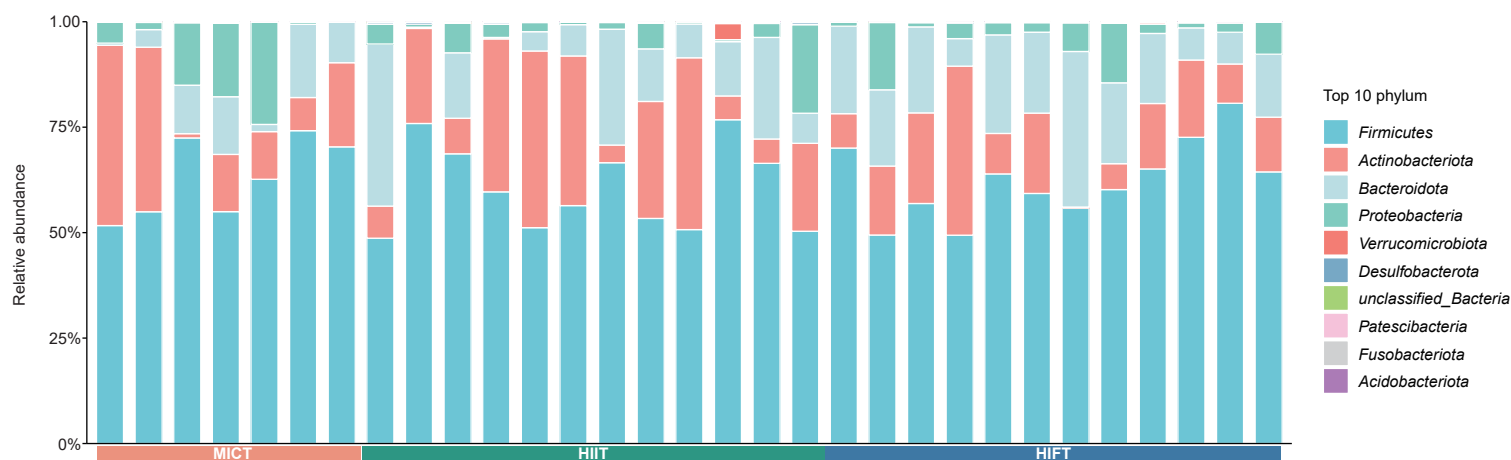

B

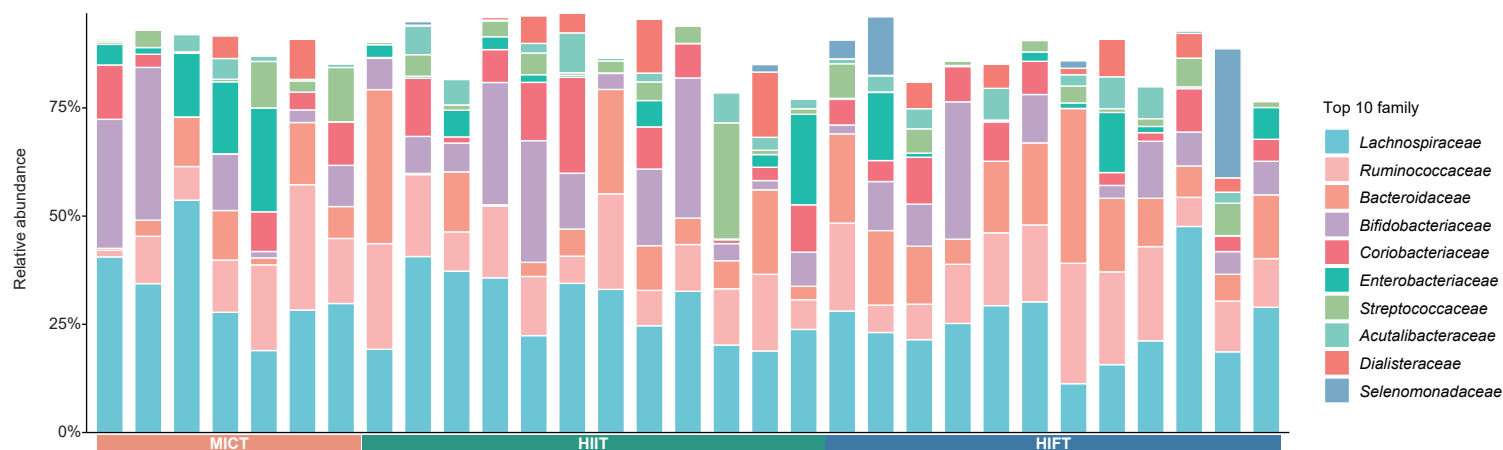

C

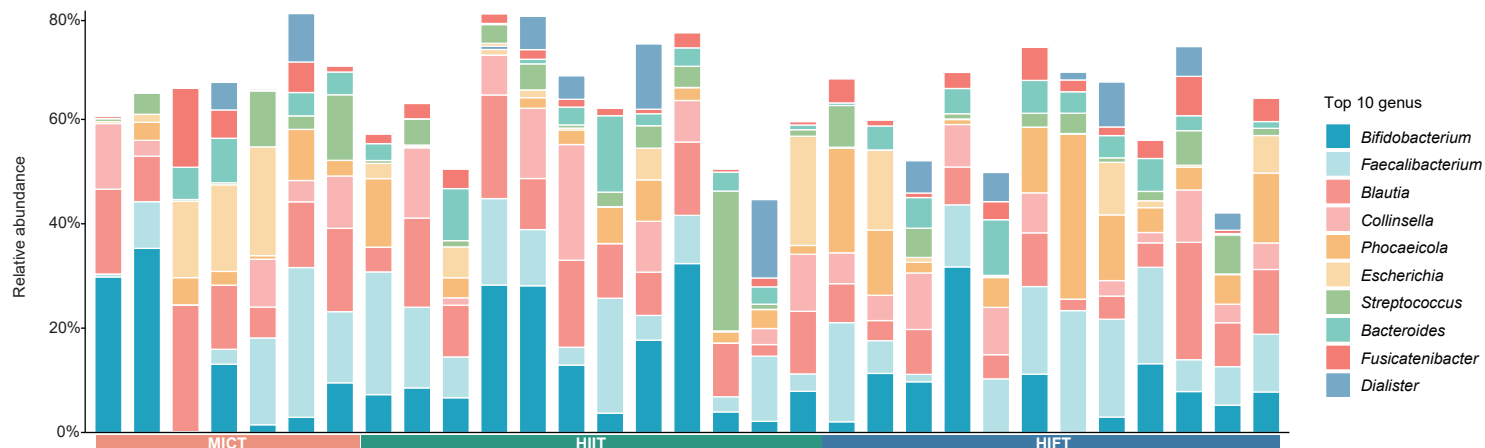

Supplement: Supplementary file 1 — Supplementary Material 1. Figure S1 An overview of the study design and participant characteristics. Figure S2 Overview for the gut microbes constitution among individuals engaged in MICT, HIIT and HIFT based on taxonomic levels. Figure S3 Distribution of bacteria at the phylum, family, and genus levels in the fecal microbiota of the current cohort comprising subjects engaged in MICT, HIIT and HIFT. Figure S4 Diverse fluctuation trend patterns of intestinal microbial general associated with various exercise modes, including MICT, HIIT and HIFT. Figure S5 Correlation of the microbial core genera KEGG pathways and enzymes and clinical parameters. Figure S6 The core enzymes specifically shift in MICT group. [file 13619_2025_244_MOESM1_ESM.zip › Figure S3.pdf]

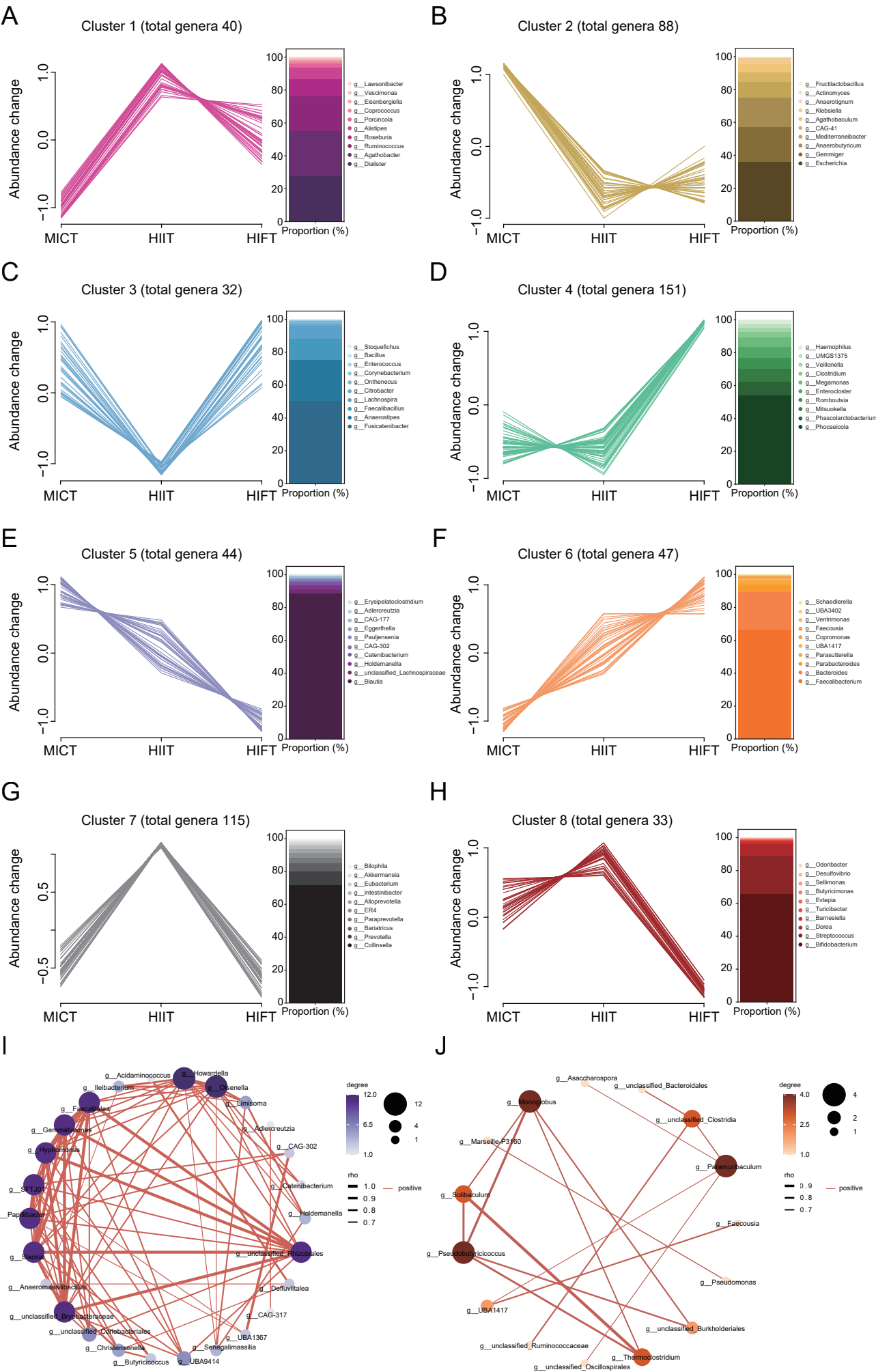

Supplement: Supplementary file 1 — Supplementary Material 1. Figure S1 An overview of the study design and participant characteristics. Figure S2 Overview for the gut microbes constitution among individuals engaged in MICT, HIIT and HIFT based on taxonomic levels. Figure S3 Distribution of bacteria at the phylum, family, and genus levels in the fecal microbiota of the current cohort comprising subjects engaged in MICT, HIIT and HIFT. Figure S4 Diverse fluctuation trend patterns of intestinal microbial general associated with various exercise modes, including MICT, HIIT and HIFT. Figure S5 Correlation of the microbial core genera KEGG pathways and enzymes and clinical parameters. Figure S6 The core enzymes specifically shift in MICT group. [file 13619_2025_244_MOESM1_ESM.zip › Figure S4.pdf]

A

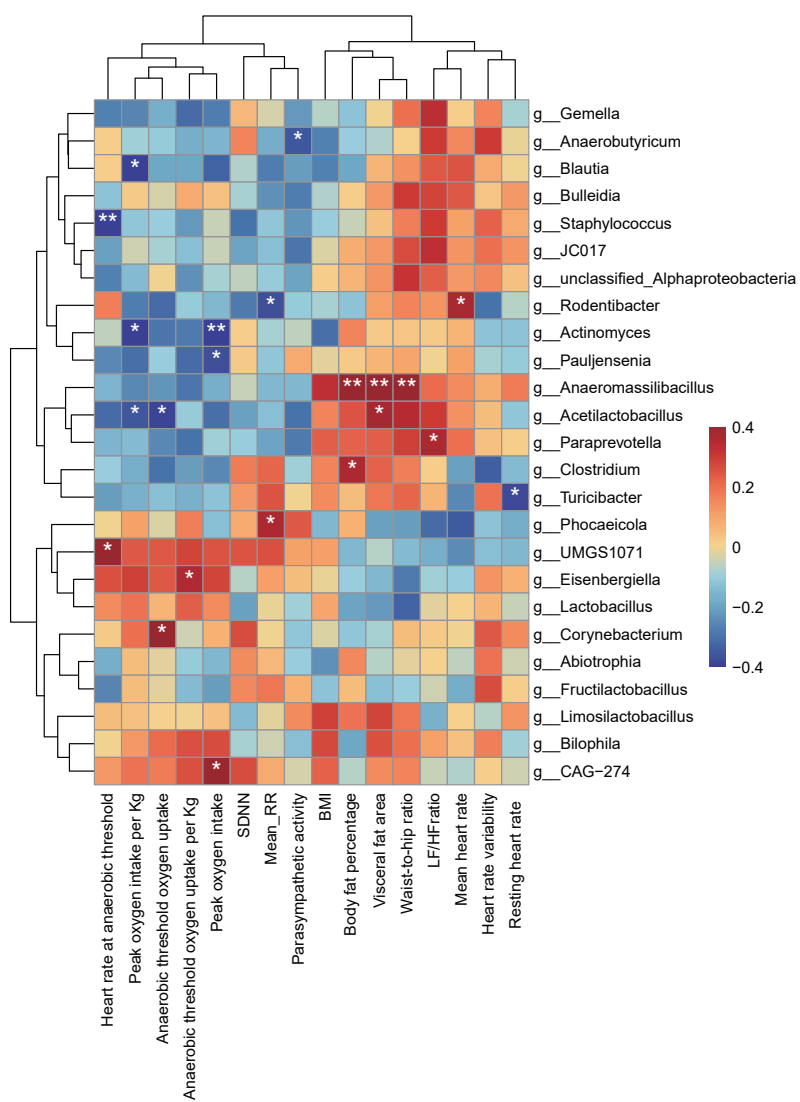

B

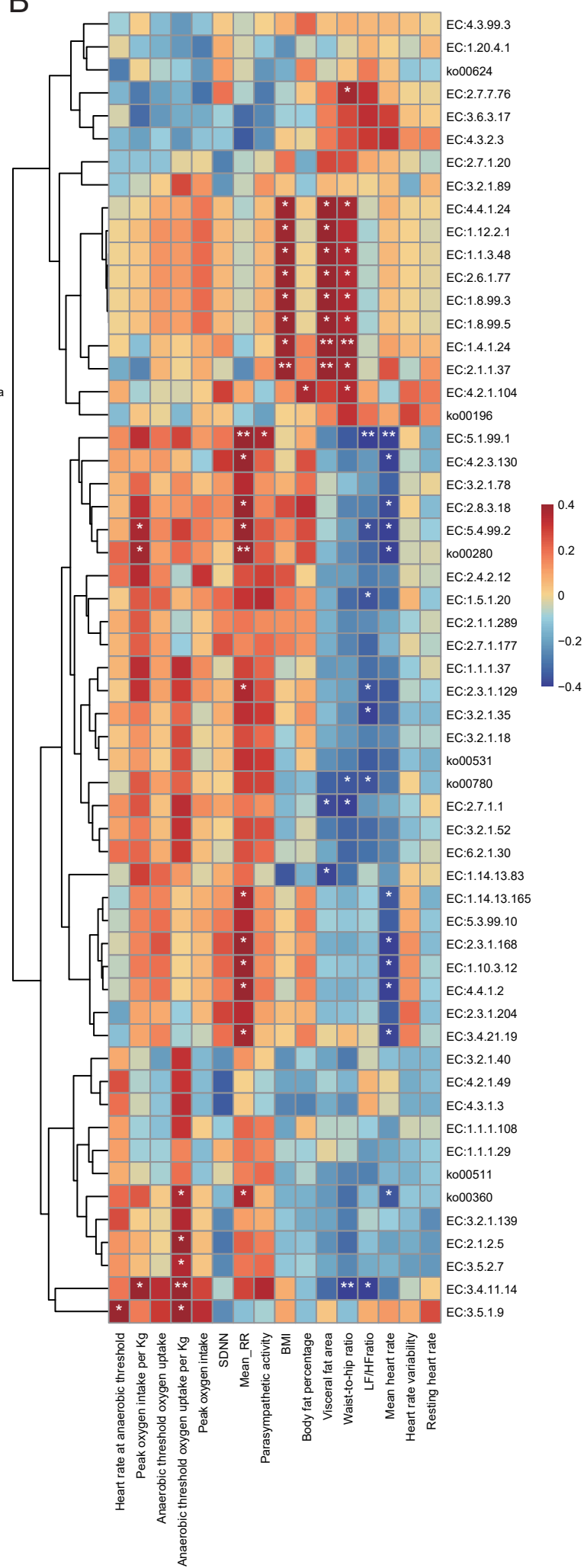

Supplement: Supplementary file 1 — Supplementary Material 1. Figure S1 An overview of the study design and participant characteristics. Figure S2 Overview for the gut microbes constitution among individuals engaged in MICT, HIIT and HIFT based on taxonomic levels. Figure S3 Distribution of bacteria at the phylum, family, and genus levels in the fecal microbiota of the current cohort comprising subjects engaged in MICT, HIIT and HIFT. Figure S4 Diverse fluctuation trend patterns of intestinal microbial general associated with various exercise modes, including MICT, HIIT and HIFT. Figure S5 Correlation of the microbial core genera KEGG pathways and enzymes and clinical parameters. Figure S6 The core enzymes specifically shift in MICT group. [file 13619_2025_244_MOESM1_ESM.zip › Figure S5.pdf]

A

45 top 16

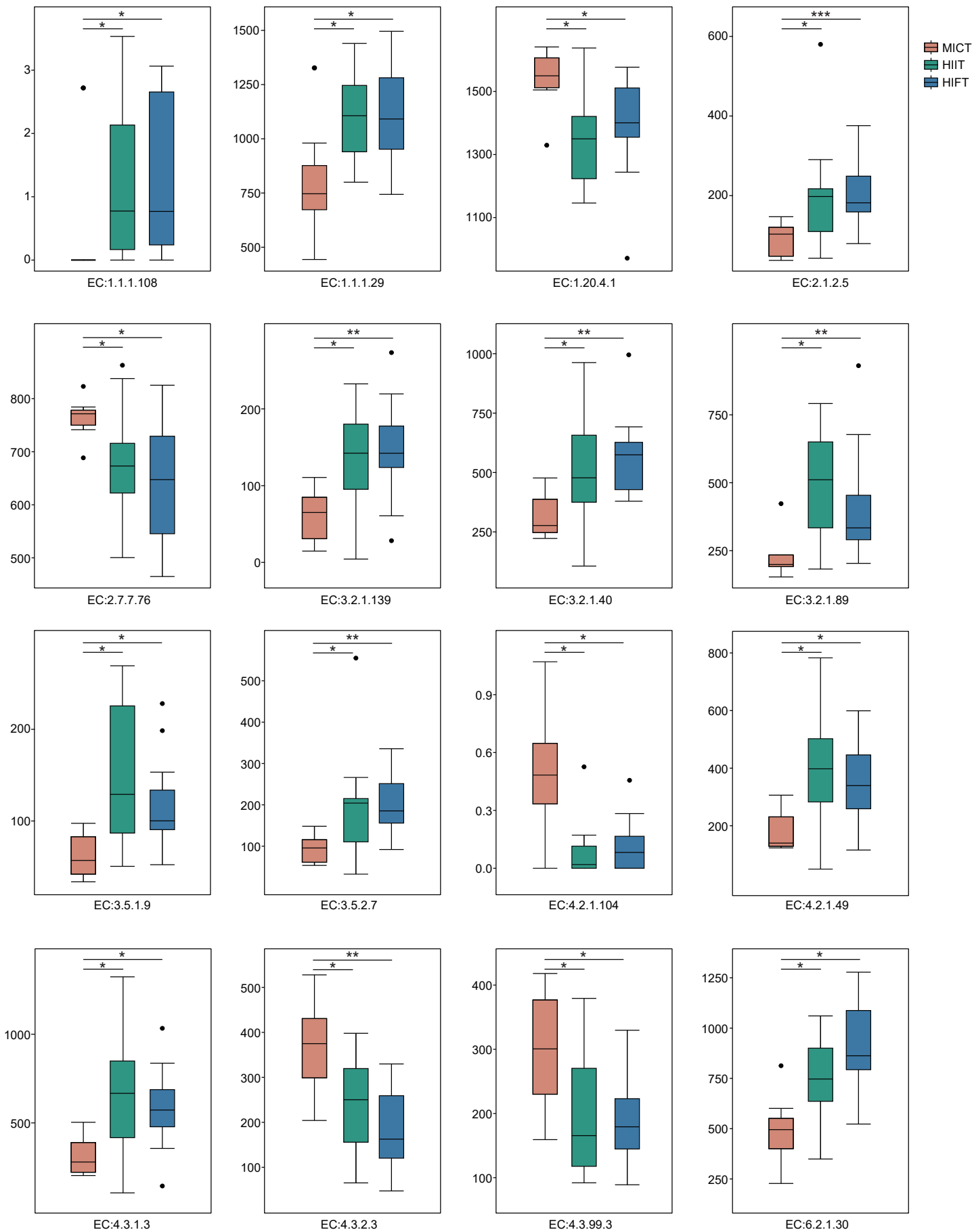

Supplement: Supplementary file 1 — Supplementary Material 1. Figure S1 An overview of the study design and participant characteristics. Figure S2 Overview for the gut microbes constitution among individuals engaged in MICT, HIIT and HIFT based on taxonomic levels. Figure S3 Distribution of bacteria at the phylum, family, and genus levels in the fecal microbiota of the current cohort comprising subjects engaged in MICT, HIIT and HIFT. Figure S4 Diverse fluctuation trend patterns of intestinal microbial general associated with various exercise modes, including MICT, HIIT and HIFT. Figure S5 Correlation of the microbial core genera KEGG pathways and enzymes and clinical parameters. Figure S6 The core enzymes specifically shift in MICT group. [file 13619_2025_244_MOESM1_ESM.zip › Figure S6.pdf]
