# Supplementary material for: Impact of exercise-induced alterations on gut microbiota diversity and composition: comparing effects of different training modalities
Source: Cell Regen. 2025 Jul 2;14:28. doi: 10.1186/s13619-025-00244-y (PMC12222581; doi:10.1186/s13619-025-00244-y)
Supplement: Supplementary file 2 — Supplementary Material 2. Table S1. Test of homogeneity of all indicators before the intervention. Table S2. General characteristics of study participants. Table S3. Details of the functional high-intensity interval training intervention. [file 13619_2025_244_MOESM2_ESM.docx]

Table S1. Test of homogeneity of all indicators before the intervention

|  |  | MICT | HIIT | HIFT | F value | P value |
| --- | --- | --- | --- | --- | --- | --- |
| Body composition | BMI (kg/m^2^) | 23.25±3.1 | 24.74±3.28 | 23±2.7 | 1.098 | 0.348 |
|  | Body fat Percentage (%) | 25.2±7.32 | 24.24±6.99 | 24.01±4.85 | 0.075 | 0.928 |
|  | Waist-to-hip ratio | 0.86±0.05 | 0.87±0.04 | 0.82±0.06 | 3.164 | 0.058 |
|  | Visceral fat area (cm^2^) | 76.57±39.67 | 83.99±29.13 | 73.63±23.12 | 0.391 | 0.68 |
| Autonomic nerve function | Mean Heart Rate (bpm) | 76.33±16.03 | 75.33±9.63 | 73.33±10.45 | 0.166 | 0.848 |
|  | Sd_HR | 2.83±1.17 | 2.92±1 | 2.17±0.84 | 2.01 | 0.154 |
|  | Heart rate variability (s) | 0.05±0.02 | 0.05±0.03 | 0.06±0.01 | 0.448 | 0.644 |
|  | Mean_RR (s) | 0.81±0.15 | 0.81±0.1 | 0.83±0.12 | 0.153 | 0.859 |
|  | SDNN | 0.04±0.02 | 0.05±0.04 | 0.05±0.02 | 0.368 | 0.696 |
|  | Sympathetic activity (ms^2^) | 0.57±0.15 | 0.53±0.13 | 0.54±0.14 | 0.179 | 0.837 |
|  | Parasympathetic activity (ms^2^) | 0.26±0.16 | 0.26±0.18 | 0.25±0.13 | 0.012 | 0.988 |
|  | Sympathovagal balance index (LF/HF ratio) | 3.15±2.46 | 3.15±2.26 | 2.55±1.05 | 0.356 | 0.704 |
| Cardiopulmonary endurance | Peak oxygen intake (mL/min) | 2.42±0.53 | 2.38±0.83 | 2.5±0.57 | 0.09 | 0.914 |
|  | Peak oxygen uptake per Kg (mL/Kg/min) | 41.7±7.45 | 39.2±5.67 | 41.28±4.15 | 0.597 | 0.558 |
|  | Peak oxygen intake per Kg (mL/Kg/min) | 33.85±6.98 | 32.63±8.55 | 30.55±6.81 | 0.438 | 0.65 |
|  | Anaerobic threshold oxygen uptake (mL/min) | 1.36±0.3 | 1.45±0.5 | 1.69±0.26 | 1.852 | 0.176 |
|  | Anaerobic threshold oxygen uptake per Kg (mL/Kg/min) | 19.23±4.79 | 20.04±5.35 | 19.75±5.77 | 0.044 | 0.957 |
|  | Resting heart rate (bmp) | 92.83±14.61 | 84.08±10.82 | 80.58±15.64 | 1.614 | 0.218 |
|  | Peak heart rate (bmp) | 173.5±40.04 | 182.42±15.96 | 158.25±42.37 | 1.569 | 0.227 |
|  | Heart rate at anaerobic threshold (bmp) | 143.83±19.77 | 142.58±12.11 | 141.17±12.78 | 0.077 | 0.926 |

Abbreviation: sd_HR, standard deviation of heart rate; mean_RR, mean RR intervals duration; SDNN, standard deviation of normal-to-normal intervals; LF, low frequency; HF, high frequency.

TableS2. General characteristics of study participants

|  |  | MICT | HIIT | HIFT | F value | P value | |
| --- | --- | --- | --- | --- | --- | --- | --- |
| Age |  | 19.02±1.67 | 19.84±2.11 | 19.56±1.83 | - | | - |
| No. |  | n=7 | n = 12 | n = 12 | - | | - |
| Body composition | BMI (kg/m^2^) | 23.07±3.22 | 24.88±3.56 | 23.33±2.59 | 0.998 | | 0.382 |
|  | Body fat Percentage (%) | 24.93±8.81 | 21.13±6.95 | 20.29±4.36 | 1.075 | | 0.355 |
|  | Waist-to-hip ratio | 0.89±0.06 | 0.88±0.04 | 0.81±0.03**## | 10.375 | | 0.000 |
|  | Visceral fat area (cm^2^) | 81.72±39.17 | 89.81±31.98 | 45.32±19.31*## | 7.528 | | 0.003 |
| Autonomic function | Mean heart rate (bmp) | 73.17±9.68 | 73.5±9.21 | 66.5±6.1 | 2.551 | | 0.097 |
|  | Sd_HR | 3±1.55 | 3.5±2.02 | 2.92±1.31 | 0.4 | | 0.674 |
|  | Heart rate variability (s) | 0.04±0.02 | 0.04±0.01 | 0.04±0.01 | 0.567 | | 0.574 |
|  | Mean_RR (s) | 0.83±0.11 | 0.83±0.11 | 0.91±0.08 | 1.982 | | 0.157 |
|  | Sd_RR | 0.04±0.01 | 0.09±0.18 | 0.04±0.01 | 0.656 | | 0.527 |
|  | Sympathetic activity (ms^2^) | 0.54±0.17 | 0.44±0.16 | 0.43±0.17 | 0.947 | | 0.401 |
|  | Parasympathetic activity (ms^2^) | 0.21±0.14 | 0.3±0.21 | 0.38±0.1 | 2.491 | | 0.102 |
|  | Sympathovagal balance index (LF/HF ratio) | 4.35±3.65 | 2.6±2.2 | 1.25±0.6** | 4.287 | | 0.024 |
| Cardiorespiratory endurance | Peak oxygen intake (mL/min) | 2.45±0.46 | 2.92±0.42 | 3.06±0.63 | 2.751 | | 0.082 |
|  | Peak oxygen uptake per Kg (mL/Kg/min) | 41.65±7.53 | 34.78±15.11 | 46.11±3.25# | 3.603 | | 0.041 |
|  | Peak oxygen intake per Kg (mL/Kg/min) | 34.25±5.75 | 36.48±8.76 | 42.78±11.5 | 2.066 | | 0.146 |
|  | Anaerobic threshold oxygen uptake (mL/min) | 1.33±0.38 | 1.44±0.46 | 1.71±0.62 | 1.355 | | 0.275 |
|  | Anaerobic threshold oxygen uptake per Kg (mL/Kg/min) | 18.42±4.3 | 20.9±4.75 | 23.46±4.63 | 2.509 | | 0.100 |
|  | Resting heart rate (bmp) | 79.83±11.62 | 83.83±15.72 | 79.42±9.4 | 0.41 | | 0.668 |
|  | Peak heart rate (bmp) | 183.67±14.62 | 183.67±16.57 | 181.67±10.29 | 0.074 | | 0.929 |
|  | Heart rate at anaerobic threshold (bmp) | 142.33±18.49 | 159.25±8.17* | 162.92±16.72** | 4.325 | | 0.023 |

Note: * indicates the significance of the difference vs. MICT group after the intervention, * indicates P <0.05; ** indicates P <0.01; # indicates the significance of the difference vs. HIIT group after the intervention, # indicates P <0.05; ## indicates P <0.01.

Abbreviation: sd_HR, standard deviation of heart rate; mean_RR, mean RR intervals duration; SDNN, standard deviation of normal-to-normal intervals; LF, low frequency; HF, high frequency.

Table S3. Details of the functional high-intensity interval training intervention

| Duration | Frequency | Exercises | Exercise Bout/Recovery Duration |
| --- | --- | --- | --- |
| 8 weeks | 3 sessions/week | Jumping Jacks | 20 s |
|  |  | Stepping | 10 s |
|  |  | High knees | 20 s |
|  |  | Stepping | 10 s |
|  |  | Side to side squat | 20 s |
|  |  | Stepping | 10 s |
|  |  | Mountain climbers | 20 s |
|  |  | Stepping | 10 s |
|  |  | Forearm plank to high plank | 20 s |
|  |  | Stepping | 10 s |
|  |  | Burpees | 20 s |
|  |  | Stepping | 10 s |
|  |  | Deep squat jumps | 20 s |
|  |  | Stepping | 10 s |
|  |  | Butt kickers | 20 s |
|  |  | Stepping | 10 s |
