# Supplementary material for: Impact of exercise-induced alterations on gut microbiota diversity and composition: comparing effects of different training modalities
Source: Cell Regen. 2025 Jul 2;14:28. doi: 10.1186/s13619-025-00244-y (PMC12222581; doi:10.1186/s13619-025-00244-y)
Supplement: Supplementary file 1 — Supplementary Material 1. Figure S1 An overview of the study design and participant characteristics. Figure S2 Overview for the gut microbes constitution among individuals engaged in MICT, HIIT and HIFT based on taxonomic levels. Figure S3 Distribution of bacteria at the phylum, family, and genus levels in the fecal microbiota of the current cohort comprising subjects engaged in MICT, HIIT and HIFT. Figure S4 Diverse fluctuation trend patterns of intestinal microbial general associated with various exercise modes, including MICT, HIIT and HIFT. Figure S5 Correlation of the microbial core genera KEGG pathways and enzymes and clinical parameters. Figure S6 The core enzymes specifically shift in MICT group. [file Figure S2.pdf › Figure S2.pdf]

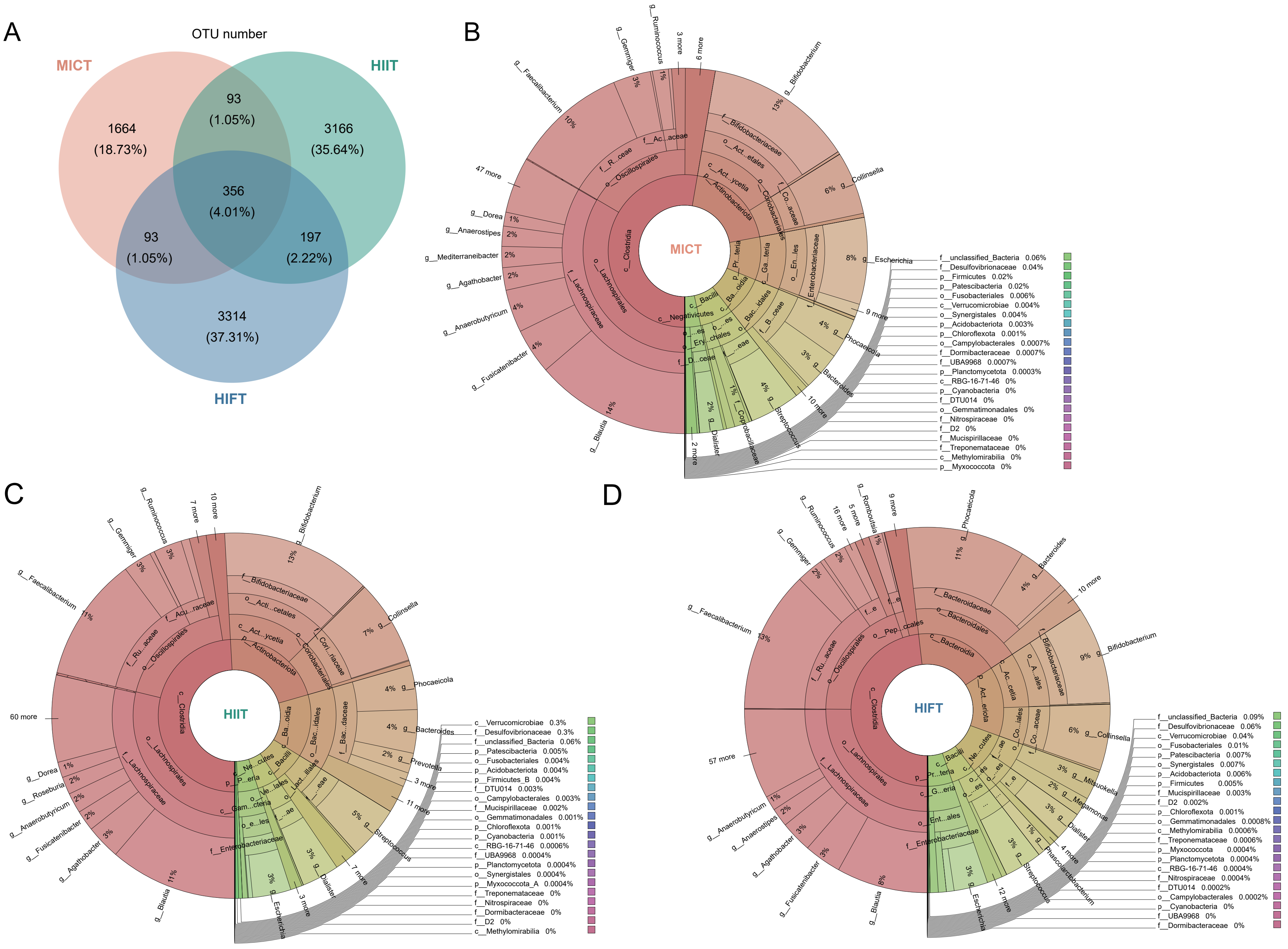

A
OTU number
MICT
HIIT
HIFT
1664
(18.73%)
93
(1.05%)
3166
(35.64%)
356
(4.01%)
93
(1.05%)
197
(2.22%)
3314
(37.31%)
B
MICT
g__Faecalibacterium 10%
g__Gemmiger 3%
g__Ruminococcus 1%
3 more
9 more
g__Bifidobacterium 13%
f__Bifidobacteriaceae
o__Act...etales
c__Act...ycetia
p__Actinobacteriota
o__Coriobacteriales
f__Co...aceae
g__Collinsella 6%
g__Escherichia 8%
f__Enterobacteriaceae
9 more
g__Phocaeicola 4%
g__Bacteroides 3%
10 more
g__Streptococcus 4%
f__Coprobacillaceae 1%
g__Dialister 2%
2 more
g__Blautia 14%
g__Fusicatenibacter 4%
g__Anaerobutyricum 4%
g__Agathobacter 2%
g__Mediterraneibacter 2%
g__Anaerostipes 2%
g__Dorea 1%
47 more
f__R...ceae
o__Oscillospirales
f__Ac...aceae
c__Clostridia
o__Lachnospirales
f__Lachnospiraceae
c__Negativicutes
f__unclassified_Bacteria 0.06%
f__Desulfovibrionaceae 0.04%
p__Firmicutes 0.02%
p__Patescibacteria 0.02%
o__Fusobacteriales 0.006%
c__Verrucomicrobiae 0.004%
o__Synergistales 0.004%
p__Acidobacteriota 0.003%
p__Chloroflexota 0.001%
o__Campylobacterales 0.0007%
f__Dormibacteraceae 0.0007%
f__UBA9968 0.0007%
p__Planctomycetota 0.0003%
c__RBG-16-71-46 0%
p__Cyanobacteria 0%
f__DTU014 0%
o__Gemmatimonadales 0%
f__Nitrospiraceae 0%
f__D2 0%
f__Mucispirillaceae 0%
f__Treponemataceae 0%
c__Methylomirabilia 0%
p__Myxococcota 0%
C
HIIT
g__Faecalibacterium 11%
g__Gemmiger 3%
g__Ruminococcus 3%
7 more
10 more
g__Bifidobacterium 13%
f__Bifidobacteriaceae
o__Acti...cetales
c__Act...ycetia
p__Actinobacteriota
o__Coriobacteriales
f__Cori...riaceae
g__Collinsella 7%
g__Phocaeicola 4%
g__Bacteroides 4%
g__Prevotella 2%
3 more
11 more
g__Streptococcus 5%
7 more
g__Dialister 3%
3 more
g__Escherichia 3%
f__Enterobacteriaceae
g__Blautia 11%
g__Agathobacter 3%
g__Fusicatenibacter 2%
g__Anaerobutyricum 2%
g__Roseburia 2%
g__Dorea 1%
60 more
f__Ru...aceae
o__Oscillospirales
f__Acu...raceae
c__Clostridia
o__Lachnospirales
f__Lachnospiraceae
c__Verrucomicrobiae 0.3%
f__Desulfovibrionaceae 0.3%
f__unclassified_Bacteria 0.06%
p__Patescibacteria 0.005%
o__Fusobacteriales 0.004%
p__Acidobacteriota 0.004%
p__Firmicutes_B 0.004%
f__DTU014 0.003%
o__Campylobacterales 0.003%
f__Mucispirillaceae 0.002%
o__Gemmatimonadales 0.001%
p__Chloroflexota 0.001%
p__Cyanobacteria 0.001%
c__RBG-16-71-46 0.0006%
f__UBA9968 0.0004%
p__Planctomycetota 0.0004%
o__Synergistales 0.0004%
p__Myxococcota_A 0.0004%
f__Treponemataceae 0%
f__Nitrospiraceae 0%
f__Dormibacteraceae 0%
f__D2 0%
c__Methylomirabilia 0%
D
HIFT
g__Faecalibacterium 13%
g__Gemmiger 2%
g__Ruminococcus 2%
16 more
5 more
g__Romboutsia 1%
9 more
g__Phocaeicola 11%
g__Bacteroides 4%
10 more
f__Bacteroidaceae
o__Bacteroidales
c__Bacteroidia
g__Bifidobacterium 9%
f__Bifidobacteriaceae
g__Collinsella 6%
g__Mitsuokella 3%
g__Megamonas 2%
g__Dialister 3%
4 more
g__Phascolarctobacterium 1%
g__Streptococcus 3%
12 more
g__Escherichia 3%
f__Enterobacteriaceae
g__Blautia 8%
g__Fusicatenibacter 3%
g__Agathobacter 3%
g__Anaerostipes 2%
g__Anaerobutyricum 1%
57 more
o__Pep...ocales
f__Ru...aceae
o__Oscillospirales
c__Clostridia
o__Lachnospirales
f__Lachnospiraceae
f__unclassified_Bacteria 0.09%
f__Desulfovibrionaceae 0.06%
c__Verrucomicrobiae 0.04%
o__Fusobacteriales 0.01%
o__Synergistales 0.007%
p__Patescibacteria 0.007%
p__Firmicutes 0.005%
p__Acidobacteriota 0.006%
f__Mucispirillaceae 0.003%
f__D2 0.002%
p__Chloroflexota 0.001%
o__Gemmatimonadales 0.0008%
c__Methylomirabilia 0.0006%
f__Treponemataceae 0.0006%
p__Myxococcota 0.0004%
p__Planctomycetota 0.0004%
c__RBG-16-71-46 0.0004%
f__Nitrospiraceae 0.0004%
f__DTU014 0.0002%
o__Campylobacterales 0.0002%
p__Cyanobacteria 0%
f__UBA9968 0%
f__Dormibacteraceae 0%
